# Genome Sequences of Two Carbapenemase-Resistant *Klebsiella pneumoniae* ST258 Isolates

**DOI:** 10.1128/genomeA.00558-14

**Published:** 2014-06-19

**Authors:** Maria Soledad Ramirez, Gang Xie, Shannon Johnson, Karen Davenport, David van Duin, Federico Perez, Robert A. Bonomo, Patrick Chain, Marcelo E. Tolmasky

**Affiliations:** a Center for Applied Biotechnology Studies, Department of Biological Science, California State University, Fullerton, Fullerton, California, USA; b IMPaM (UBA-CONICET), Buenos Aires, Argentina; c Los Alamos National Laboratory, Los Alamos, New Mexico, USA; d Louis Stokes Cleveland Department of Veterans Affairs Medical Center, Cleveland, Ohio, USA; e Department of Medicine, Case Western Reserve University School of Medicine, Cleveland, Ohio, USA; f University of North Carolina, Chapel Hill, North Carolina, USA

## Abstract

*Klebsiella pneumoniae*, an ESKAPE group (*Enterococcus faecium*, *Staphylococcus aureus*, *Klebsiella pneumoniae*, *Acinetobacter baumannii*, *Pseudomonas aeruginosa*, and *Enterobacter* species) pathogen, has acquired multiple antibiotic resistance genes and is becoming a serious public health threat. Here, we report the genome sequences of two representative strains of *K. pneumoniae* from the emerging *K. pneumoniae* carbapenemase (KPC) outbreak in northeast Ohio belonging to sequence type 258 (ST258) (isolates Kb140 and Kb677, which were isolated from blood and urine, respectively). Both isolates harbor a *bla*_KPC_ gene, and strain Kb140 carries *bla*_KPC-2_, while Kb677 carries *bla*_KPC-3_.

## GENOME ANNOUNCEMENT

*Klebsiella pneumoniae*, a bacterium belonging to the ESKAPE group (*Enterococcus faecium*, *Staphylococcus aureus*, *K. pneumoniae*, *Acinetobacter baumannii*, *Pseudomonas aeruginosa*, and *Enterobacter* species), is responsible for serious community- and hospital-acquired infections (1–5). The difficulty in treating infections caused by *K. pneumoniae* is increasing due to its ability to acquire antibiotic resistance genes (6–8), including carbapenemases (9).

Despite the clinical importance of this bacterium, the number of identified and characterized virulence factors has remained relatively low (10–13). Nucleotide and whole-genome mapping comparisons among several strains led to the identification of a high heterogeneity zone (HHZ) (14), which includes the capsular polysaccharide biosynthesis gene cluster (HHZ subregion 4) and a hot spot (HHZ subregion 3), which in strain *K. pneumoniae* NTUH-K2044 is the point of insertion of a fragment that includes a pathogenicity island related to that found in *Yersinia* species. This fragment includes the genes coding for the yersiniabactin siderophore system, a region previously identified in a *Klebsiella* plasmid, and genes coding for conjugation functions (10, 14, 15).

*K. pneumoniae* strains harboring carbapenem resistance genes, such as *bla*_KPC_ or *bla*_NDM-1_, are becoming more frequent and extremely problematic. *K. pneumoniae* Kb140 and Kb677, two representative strains from an emerging KPC outbreak in northeast Ohio, were isolated in 2012. Kb140 was isolated from a patient with fatal bloodstream infection and pneumonia, while Kb677 was isolated from a patient with urinary tract colonization. Both strains were draft sequenced using Illumina (353× and 293× genome coverage for Kb140 and Kb677, respectively) and PacBio RS II (100× and 105× genome coverage, respectively). The submitted *de novo* assemblies utilized Velvet (version 1.2.08), Newbler (version 2.6), AllPaths (version 44837), HGAP (version 2.1.1), and parallel Phrap (SPS-4.24), along with manual review and curation. The draft genomes of Kb140 and Kb677 consist of 5,677,714-bp and 5,894,762-bp sequences, respectively.

There are 5,420 and 5,499 predicted protein-coding genes within the genomes of *K. pneumoniae* isolates Kb140 and Kb677, respectively. Of these, 20.7% and 21.9% of the protein-coding genes, respectively, are annotated as hypothetical or conserved hypothetical proteins. Of those with functional predictions, 31 (Kb140) and 68 (Kb677) are associated with phages/prophages, while 103 (Kb140) and 126 (Kb677) are associated with resistance to antibiotics or toxic compounds. The *bla*_KPC_ genes are likely located within the sequence with accession no. AQRD01000007 of the Kb140 assembly (positions 1431915 to 1432775) and the sequence with accession no. AQPG01000002 of the Kb677 assembly (positions 7281 to 8240), respectively. The strain Kb140 and Kb677 genomes have 86 and 85 tRNA genes, respectively, as well as 24 rRNA genes.

*K. pneumoniae* strains usually harbor several plasmids (4, 6, 16). Analysis of the nucleotide sequences of strains Kb140 and Kb677 showed homology to the IncFII_k_-FIB-like plasmids pKPN- and pKpQIL-type (17, 18), pIncX-SHV (17), and pKP1780-kpc (accession no. KF874497), pNJST258C1, pNJST258C2 (19), pBK31551, pBK15692 (20), p1 (accession no. CP006657), pR55 (21), and the *K. pneumoniae* subsp. *rhinoscleromatis* pKRH (22).

### Nucleotide sequence accession numbers.

The GenBank accession no. for *K. pneumoniae* Kb140 and Kb677 are AQRD01000001 to AQRD01000008 and AQPG01000001 to AQPG01000012, respectively.
